# Humanized Mice for Live-Attenuated Vaccine Research: From Unmet Potential to New Promises

**DOI:** 10.3390/vaccines8010036

**Published:** 2020-01-21

**Authors:** Aoife K. O’Connell, Florian Douam

**Affiliations:** Department of Microbiology, National Emerging Infectious Diseases Laboratories, Boston University School of Medicine, Boston, MA 02118, USA; aocon@bu.edu

**Keywords:** animal model, bacterial vaccine, humanized mice, immune response to vaccine, immunogenicity, live-attenuated vaccine, vaccine, viral vaccine

## Abstract

Live-attenuated vaccines (LAV) represent one of the most important medical innovations in human history. In the past three centuries, LAV have saved hundreds of millions of lives, and will continue to do so for many decades to come. Interestingly, the most successful LAVs, such as the smallpox vaccine, the measles vaccine, and the yellow fever vaccine, have been isolated and/or developed in a purely empirical manner without any understanding of the immunological mechanisms they trigger. Today, the mechanisms governing potent LAV immunogenicity and long-term induced protective immunity continue to be elusive, and therefore hamper the rational design of innovative vaccine strategies. A serious roadblock to understanding LAV-induced immunity has been the lack of suitable and cost-effective animal models that can accurately mimic human immune responses. In the last two decades, human-immune system mice (HIS mice), i.e., mice engrafted with components of the human immune system, have been instrumental in investigating the life-cycle and immune responses to multiple human-tropic pathogens. However, their use in LAV research has remained limited. Here, we discuss the strong potential of LAVs as tools to enhance our understanding of human immunity and review the past, current and future contributions of HIS mice to this endeavor.

## 1. Introduction

Live-attenuated vaccines (LAVs) have saved millions of lives and continue to prove themselves as one of the most important inventions in modern medical history. Smallpox was successfully eradicated in 1980 using the closely-related vaccinia virus [1], and live measles vaccines averted an estimated 21.1 million deaths between 2010 and 2017 [2]. The immunogenicity of LAVs is superior to other vaccine types: inactivated, subunit, and toxoid vaccines may require additional doses and adjuvants to achieve s–ufficient immunity (Table 1) [3]. In contrast, a single dose of yellow fever vaccine provides protection against the disease for at least ten years [4]. While some concerns remain about the ability of LAVs to revert toward a wild-type genotype in humans, the adverse effects and risks associated with these vaccines remain very low and are clearly outweighed by the health benefits they provide at a global scale. Over recent years, alarming episodes of pathogen (re-) emergence for which there are no licensed vaccines, such as Ebola, Lassa, or Zika virus, have been witnessed [5,6,7]. Additionally, the emergence potential of currently neglected pathogens such as Powassan virus and Eastern equine Encephalitis Virus [8] strongly highlight the global need for better preparedness against future epidemics. 

A primary limitation in developing innovative vaccines against today and tomorrow’s challenging infectious diseases is our inability to understand the molecular basis of current LAV attenuation and immunogenicity. This is mainly because of the empiric nature of their development [9]. Traditionally, LAV attenuation has been achieved either through the serial passage of a virulent pathogen or the isolation of a closely related virus or bacterium that produces mild disease in humans, such as cowpox for smallpox vaccines [9]. This means that the existence of a LAV directly correlates with the capacity of its parental or common ancestor strain to evolve and adapt to a novel host or environment. Therefore, LAV attenuation is tied to the stochasticity of evolution. The story of the generation of the yellow fever vaccine strain 17D strongly supports such reasoning. 17D was isolated by serially passaging a virulent yellow fever virus (YFV) strain in mouse and chicken embryo tissues [10]. Despite several attempts to repeat the process, 17D could not be isolated more than once [10]. 

A major roadblock in our quest to understand mechanisms of LAV attenuation and immunogenicity is the absolute need to study human-specific immune responses in vivo and in controlled experimental settings. When studying LAV responses in a cohort of voluntary human vaccinees, peripheral blood can be readily assessed, yet tissues from the site of infection or from secondary lymphoid organs where the immune response is primed cannot. Additionally, to precisely pinpoint the attenuation mechanisms of a LAV, it is important to perform a direct comparison between the immune responses induced by LAVs and by their virulent counterpart. However, such a comparison, as well as the genetic manipulation of the immune system, remains ethically impossible in human patient cohorts. Therefore, our understanding of LAV attenuation and immunogenicity fully relies upon the identification of a cost-effective and easily accessible animal model that can accurately recapitulate human immune responses upon vaccination.

Humanized mice, or immunodeficient mice harboring human tissues and/or genes, have emerged as viable preclinical models for modeling human biological process and disease [11]. Mice engrafted with human immune system (HIS mice) components have notably been instrumental in studying the infectious cycle of human-tropic bacteria and viruses in vivo and understanding how these pathogens interact with the human immune system [12,13]. However, the contributions of HIS mice to LAV research has remained limited in comparison to other model systems, such as the human model or non-human primate (NHPs) models, mainly because conventional HIS mice harbor important limitations for accurately modeling LAV-induced immunity, such as limited human hematopoiesis, improper immune priming and hampered adaptive response. Over the past five years, novel HIS mice have been developed and now demonstrate a superior ability to mount potent innate and adaptive immune responses against immunogens and pathogens [14,15,16,17,18]. These emerging models, and future ones to come, offer unprecedented opportunity to better understand how LAVs interact with the human immune system over time and space, at the site of infection and in secondary lymphoid tissues.

This review will encompass the past, current and future applications of HIS mice for elucidating the molecular mechanisms governing LAV attenuation and immunogenicity. First, we will discuss the empiric development of several historically important LAVs and how they have positively impacted global human health, and review some of the molecular mechanisms suspected to underly their attenuation. Next, we will provide an overview of the past and current HIS mouse models and explore the contributions of HIS mice to our understanding of LAV attenuation and immunogenicity. Finally, we will identify the shortcomings associated with these models and describe how current and future efforts in refining HIS mice models will open avenues for a better understanding of LAV-induced immunity. 

## 2. Live-Attenuated Vaccines

For over 200 years, LAVs have served as invaluable tools for controlling and/or eradicating infectious diseases. The idea that infection by live pathogens can confer protection against deadly infectious diseases has been employed throughout history through procedures such as variolation [19,20,21,22,23,24]. However, by the end of the 18th century, such an idea was popularized by Edward Jenner who demonstrated that inoculation with the cowpox virus, which only produced mild illness in humans, provided immunity against smallpox, a deadly disease induced by a closely related virus, the variola virus [25]. With this discovery, Jenner not only promoted the concept of live-attenuated vaccines, but the general concept of vaccination as well, an idea that would save hundreds of millions of lives in the following centuries. 

In the 1800s, research by Louis Pasteur with chicken cholera [26], anthrax [27], and rabies [28] introduced the concept that LAVs could be generated by attenuating deadly pathogens through exposure to heat or oxygen. During the early 20th century, the serial passage of pathogens in specific media was also found to be an effective way to attenuate pathogens and generate LAVs. The tuberculosis Bacille Calmette–Guérin (BCG) vaccine was the first vaccine to be attenuated through serial passages. Formulated by the repeated subculture of *Mycobacterium bovis*, the bacterium responsible for bovine tuberculosis, the generation of the BCG vaccine therefore successfully integrated the vaccination concepts described by Jenner and Pasteur [29]. By being made through the attenuation of pathogens related to the bacterium or virus responsible for a disease, the BCG vaccine hence represents what could be considered a second category of LAVs. The pentavalent rotavirus vaccine is another LAV of this category, which contains a combination of attenuated human and bovine rotavirus strains [30].

The advent of pathogen attenuation through serial passage brought forth the most predominant category of LAVs, which are made directly through attenuation of the causative pathogen of a disease. Serial passage was performed in animal hosts, embryonated eggs, and eventually in tissue culture [31]. Some of the most notable, established LAVs generated this way are the yellow fever [32], poliovirus [33], measles [34], mumps [35], rubella [36], typhoid [37], varicella [38], and zoster [39] vaccines.

LAVs undeniably represent the most effective class of vaccines ever developed. Based on the success of past LAVs, several LAV candidates targeting challenging pathogens such as Dengue virus [40], Japanese Encephalitis virus [41], West Nile virus [42], Zika virus [43], SARS [44], Malaria [45] or Ebola virus [46,47], have been designed and evaluated. For instance, in response to the Ebola virus disease outbreaks in Africa between 2013 and 2016, a recombinant vesicular stomatitis virus (VSV) expressing the envelope glycoprotein of Ebola virus was generated (VSV-EBOV). VSV induces asymptomatic infection in humans and causes only mild, transient illness, and the vaccine has demonstrated safety and immunogenicity in multiple phase I and II/III clinical trials [46,47]. 

Unlike other types of vaccines, LAVs carry both potent immunogenic epitopes and the ability to replicate, at a highly tuned rate, into their host. Therefore, their effectiveness relies primarily upon a very fine control of the virus’s ability to replicate: LAVs have to replicate enough for immunogenic epitopes to spread and activate a potent immune response, but at a limited and stable enough rate to prevent adverse effects or genetic reversion toward a wild-type phenotype, as well as to allow subsequent immune control of infection (Table 1).

The methods by which established LAVs have been generated (use of related pathogens, attenuation of related pathogens, and attenuation of the causative agent of a disease), are all empiric and were conducted without any knowledge of the immunological mechanisms that are at play during LAV infection. Specific mutations in viral or bacterial genomes, which likely translate into differential host–pathogen interactions, have been clearly associated with pathogen attenuation. However, the nature of these specific host–LAV interactions, and how they trigger a potent immune response, remain mostly unknown. The emergence of new infectious diseases, minimization of adverse reactions to LAV and reversion to wild-type strains, short-tracking of vaccine production methods, and provision of immunization options for immunocompromised individuals, are only a few of the reasons why researching the mechanisms governing LAV attenuation still remain of critical importance today. Despite the development of potentially promising LAV candidates over the past two decades to fight many challenging infectious diseases (see examples above), a very limited number of them have actually been licensed so far, suggesting that novel rational approaches are needed to design more potent and/or safer LAVs in the future. To do so, the currently established LAVs represent a formidable source of information. In this first section, we will review the historical development and global impact of established LAVs, and discuss some of the attenuation mechanisms that have been identified for these vaccines. 

### 2.1. Using Closely Related Pathogens as LAVs: The Smallpox Vaccine 

Smallpox was a deadly disease that plagued humanity for thousands of years until its eventual eradication in 1980 [1,48]. The disease was caused by the virus variola from the *Orthopovirus* genus and poxvirus family, and had a case fatality rate of 30% [49]. Variolation, or infection with material from smallpox pustules or scabs, was practiced in China and India for thousands of years before its introduction into Europe in the 1790s [19,20,21,22,23,24,50]. Although variolation was safer than the contraction of smallpox itself, it had a 1% chance of mortality even if executed correctly [9]. While it was commonly known that those who contracted cowpox (a zoonotic poxvirus closely related to variola that only induced mild disease in humans) did not contract smallpox, deliberate inoculation with the virus was not popularized until the experiments of Edward Jenner were published in 1798. Jenner’s findings summoned a wave of enthusiasm for what would eventually be termed ‘vaccination’ but issues with using cowpox as a LAV, which included bacterial contamination and unmeasured potency, caused vaccination support to wane. Early vaccines were made of lymph from infected calves or were propagated between vaccinees, but arm-to-arm transfer was considered unsafe because of the spread of bloodborne diseases [49]. A virus closely related to cowpox and variola known as vaccinia took the place of cowpox as the source of smallpox LAVs by 1900 through what may have also been contamination or the result of varied methods of virus passage [51]. 

Despite initial setbacks in smallpox vaccination, vital scientific advancements were made in the 20th century, including limitations on vaccine bacterial count (i.e., non-pathogenic bacterial contamination of vaccine preparation), determination of the required potency to induce smallpox immunity [51], and the introduction of a seed lot system by the World Health Organization in 1959, which created a vaccine supply of greater consistency [52]. Temperature stabilization through freeze drying [53] and the invention of a bifurcated needle [54] allowed the vaccine to be transported over greater distances and administered more efficiently. LAV enhancements, along with the lack of an animal reservoir for the disease, made smallpox a prime candidate for global eradication. For these reasons, the World Health Organization (WHO) initiated the Smallpox Eradication Program in 1959 [51]. Many vaccine strains were used in this program, but some of the most commonly used LAVs were Dryvax and EM-63, derived from the NYCBH strain, Elstree of the Lister strain, and Tiantan of the Temple of Heaven Strain [51]. Through global effort, and with the aid of effective LAVs, smallpox was eradicated by 1980 [1].

Vaccinia LAVs are potent but reactogenic, and complications are very uncommon but can be serious [55]. They are also unsuitable for immunocompromised individuals who would be most at risk in the case of an epidemic [56]. In light of recent outbreaks of known and novel zoonotic poxviruses [57,58,59,60] and the potential use of smallpox in bioterrorism [51], second-generation vaccines such as ACAM2000 [61] and CJ-50300 [62] have been derived from original vaccinia strains in tissue culture, and other LAVs have been made from the highly attenuated, modified vaccinia Ankara strain [63,64]. Further understanding of the interaction of vaccinia LAV with the human immune system will be a major part of developing new LAVs to combat *Orthpoxviruses* in the future.

### 2.2. Generating LAV through Pathogen Attenuation

Following Jenner’s success with smallpox vaccines, Louis Pasteur made strides that initiated a novel generation of LAV. This advancement was the attenuation, or weakening, of the pathogens responsible for disease. After finding that cultures of *Pasteurella multocida*, the bacterium of chicken cholera, were attenuated when left out and exposed to air, Pasteur procured a vaccine from this [26]. He proceeded to produce an anthrax vaccine in animals, whose etiological agent had been discovered by Robert Koch [27]. Pasteur’s contribution to human LAV came to fruition through his work on rabies, which he attenuated though drying the spinal cord of infected rabbits [28]. Through a regimen of inoculation with progressively less dried (and more virulent) samples, rabies could be prevented and treated. In 1885, Pasteur’s attenuated rabies vaccine was successfully administered to a human patient, establishing itself as the first truly attenuated human LAV [28]. This LAV incorporated wisdom from both Jenner and Pasteur’s research in its production and offered protection from one of the deadliest pathogens in existence. 

#### 2.2.1. Attenuation of Related Pathogens: The Tuberculosis Vaccine

Tuberculosis (TB) is a bacterial infection caused by *Mycobacterium Tuberculosis.* This bacteria has caused more deaths than any other infectious pathogen, although treatment and prevention methods exist [65]. Out of an estimated 1.7 billion individuals who are currently infected with *M. tuberculosis*, 5–10% will develop active TB and will transmit the bacteria to 10 to 15 individuals each year [66]. Therefore, a vaccine against TB has been, and still remains, essential to contain this infectious disease. Today, the only licensed TB vaccine available is the Bacille Calmette–Guérin (BCG) LAV [67].

*M. tuberculosis* was first identified by Robert Koch in 1882 [68], although the bacterium used in the BCG LAV is *Mycobacterium bovis*, a pathogen responsible for bovine tuberculosis, and one that is equally virulent in humans. The attenuation of *M. bovis* was achieved by Albert Calmette and Camille Guérin of the Institut Pasteur, who subcultured an isolation of *M. bovis* in media containing potato, glycerol, and beef bile for 13 years and a total of 231 serial passages [69]. The attenuated BCG vaccine was successfully used on an infant in 1921 [70]. Apart from an *M. tuberculosis* contamination that resulted in the Lübeck disaster in 1930, which killed 72 infants and infected an additional 135 of 250 infants vaccinated [71], BCG administration became widespread without ill effect, and multiple strains were thus developed [70]. 

Apprehension regarding TB following World War II led multiple health organizations to advocate for the BCG vaccine [70]. While its efficacy had been proven by earlier studies, it became evident that it varied in effectiveness in different areas of the world [70]. Consequently, the WHO introduced a seed lot system for LAV uniformity in 1956 and, in the countries reporting to the WHO, more than ten BCG strains are currently in use [67]. In recent years, the immunogenicity of the BCG LAV has been studied to a greater extent and appears to vary based on age at the time of vaccination, location, and previous exposure to mycobacteria or infection [72,73,74,75]. Statistical analyses of efficacy studies show that the BCG vaccine is most effective in providing protection against severe extrapulmonary (non-lung) forms of pediatric TB, but is unreliable in its protection of adults against pulmonary TB [72]. However, despite its variable efficacy, the BCG vaccine has been incredibly beneficial, and prevented an estimated 45 million deaths in HIV-negative individuals and 9 million HIV-positive individuals between 2010 and 2017 alone [65].

TB remains one of the top ten causes of death in the world, and the source of major epidemics in developing countries [65]. Additionally, HIV-positivity is a contraindication for BCG vaccination, although these individuals are exponentially more likely to develop active TB [56,65]. Another threat to the eradication of TB is the increasing prevalence of multidrug-resistant tuberculosis (MDR-TB) and extensively drug-resistant TB (XDR-TB) [65]. Efforts have continued to reach milestones that were established by the WHO’s End TB strategy, but the current trajectory of TB decline will not meet previous deadlines [65]. Although BCG LAV plays an essential role in eradicating TB, new and effective vaccine strategies are now urgently needed. 

#### 2.2.2. Attenuation of Virulent Pathogens

Calmette and Guérin’s use of serial passage to develop an LAV opened new doors in vaccine production. The majority of LAVs have since been made using this method, including vaccines against yellow fever [32], poliomyelitis [33], measles [34], mumps [35], rubella [36], typhoid [37], varicella [38], zoster [39], and influenza [76]. Rotateq, a pentavalent reassortant rotavirus vaccine, was made with a strategy like that of BCG vaccines, and is comprised of a combination of attenuated human and bovine strains of the virus [30]. Techniques used in serial passage have evolved over time, occurring in a variety of media and host organisms. The effect these LAVs have had on disease prevention has been profound. In 1951, Max Theiler was awarded a Nobel Prize for his creation of the yellow fever LAV [77], and remains the only recipient of such an award for the development of a vaccine [78]. We will summarize the history of some of the most well-known LAVs of this category, including yellow fever, measles, polio, and influenza vaccines.

##### Yellow Fever

Yellow fever (YF) is a serious hemorrhagic fever endemic to tropical and subtropical regions of Africa and South America [79]. It is recognized as one of the most devastating infectious diseases of the 17th and 18th century, capable of killing thousands at a time [80,81]. YF is caused by yellow fever virus (YFV), a positive-sense RNA flavivirus, and is transmitted by *Aedes, Haemogogus*, and *Sabethes* mosquito species [82]. The first isolations of YFV were obtained through serial passage of the serum of infected individuals, Francois Mayali and Asibi, in rhesus macaques [83,84]. The isolation from Mayali was used by the Pasteur Institute to develop the French Neurotropic Vaccine strain (FNV), but was discontinued in 1982 due to a number of encephalitic reactions to the vaccine [10]. The YFV-Asibi strain was isolated and passaged by Max Theiler of the Rockefeller Institute, and was successfully attenuated in 1927 after 235–240 passages in mouse embryonic tissue, minced whole-chick embryo, and minced chick embryo with brain and spinal cord removed [32]. This LAV caused mild disease in macaques and human cohorts and conferred protective immunity against YFV [85]. This strain is known as YFV-17D and is the basis of all YFV vaccines used today [86].

Mass vaccination campaigns in Africa and South America have succeeded in controlling yellow fever in endemic areas over the past 80 years. However, recurrent outbreak episodes have taken place in areas with limited vaccination coverage since the introduction of YFV-17D, with dramatic consequences at times such as in Ethiopia in 1961, where over 100,000 cases and 30,000 fatalities were reported [82]. YFV-17D and derived LAVs are renowned for their safety, potency, and immunogenic properties. YFV-17D vaccines provide effective immunity in 80–100% of vaccinees 10 days after vaccination, and more than 99% of vaccinees within 30 days of vaccination [87,88]. The incidence of serious adverse events, such as vaccine-associated neurotropic disease (YEL-AND) and vaccine-associated viscerotropic disease (YEL-AVD), remains extremely low [9]. YFV-17D appears to truly be a product of stochastic generation, as attempts to recreate it have been futile [10]. 

The diminishing supply of YFV-17D LAV in the face of unusual YF outbreaks is currently a cause for concern. Since 2016, cases of YF in nonendemic areas or areas with less prevalence of YF have increased [82]. The reason for the spread of YFV to these areas is unknown, but could be the result of increased human activity in ecosystems where sylvatic cycles of YFV transmission occur, or due to a widening range of favorable habitats for YFV-carrying mosquitoes to travel due to climate change [82]. With no antiviral treatment in existence for YF, and an existing reservoir for the disease in nonhuman primates, it is critical that vaccination in areas experiencing YF outbreaks remains consistent [79]. However, the shortage of YFV-17D vaccines has led to the use of factional doses of vaccine. These doses (ranging between 1:2 to 1:170 of the original dose) can induce neutralizing antibodies that remain detectable from one to up to eight years post vaccination [89]. It is unclear, however, whether the long-term immunity lesser doses provide will be comparable to full LAV doses at a global scale. As YF reemerges in nonendemic areas, effective surveillance protocols and adequate vaccine stockpiles will be key for preventing future outbreaks.

##### Measles

Measles is a highly contagious illness produced by the negative-sense RNA measles virus (MV) of the family *Paramyxoviridae*, and is transmitted through respiration or aerosol [90]. Prior to the licensure of a vaccine in 1963, measles epidemics occurred every two to three years and caused 2.6 million deaths annually [91]. MV was first isolated in tissue culture by Enders and Peebles in 1954 using samples from an infected child named David Edmonston [34]. The virus was propagated on primary human kidney and amnion cells and attenuated through passages in chick embryos and chick embryo fibroblasts. This process gave rise to Edmonston A and B strains [34,90]. While Edmonston A was too reactogenic for vaccination use, Edmonston B was licensed as an LAV in 1963, with further attenuation through five additional passages on chick embryo fibroblasts at 36–37 °C [9]. The reactogenicity of measles LAV (MV-LAV) led to the creation of further attenuated strains, such as AIK-C from the original Edmonston strain [92], Schwarz from Edmonston A [93], and Edmonston Zagreb [94] and Moraten [95] from the Edmonston B strain. Approaches to developing these strains involved further passage in chick cell culture [93], passage in chick cell culture at elevated [95] or lowered temperatures [92], and passage in human diploid fibroblasts [94]. Independent measles strains such as CAM 70 [96], Leningrad 4 [97], Changchun 47 and Shanghai 191 [98] were also produced. All MV-LAV cause fevers and a rash to some extent, but most side effects are mild and short-lived [9]. Safe, effective, and affordable MV-LAVs have had a profound effect on the disease: between 2000 and 2017, the incidence of measles decreased by 83%, and deaths due to measles fell by 80% [99]. 

Although a potent, cost effective, and reliable MV-LAV is available, gaps in vaccination coverage have led to its resurgence in many areas of the world. In 2017, 110,000 deaths from measles were recorded [99], and the incidence of measles has continued to rise. The high communicability of MV necessitates a high level of global vaccination coverage, and the recent pattern of outbreaks due to vaccine hesitancy have the potential to undo decades of work invested into controlling this infectious disease. The Global Vaccine Action Plan aimed to eradicate measles (and rubella) in five WHO regions by 2020 [100], but MV-LAVs must be extensively utilized to accomplish any eradication in a timely manner. 

##### Polio

Poliovirus LAVs have brought the once-feared paralytic disease, poliomyelitis, to the brink of eradication. Poliomyelitis became prevalent in the turn of the 20th century, instigating epidemiological studies that helped to characterize the virus and its pathogenesis [101]. The pathogenic theory of poliovirus was disrupted by a purely neurological view (due to the motor neuron infection that results in the disease of poliomyelitis) [9], but this was disproved by Sabin and other scientists in the 1950s [102,103,104]. Complete immunity to poliovirus requires vaccination with LAV derived from its three serotypes [101]. The LAVs used against poliovirus are derived from the Sabin type 1, 2, and 3 vaccine strains developed in 1954, and originate from parental strains Mahoney, P712, and Leon, respectively [33]. The attenuation of these strains was achieved through tissue culture passage in a variety of cell types as well as intracerebral passage in the case of P712 [9,105]. Following serial passage of each serotype, Sabin selected the most successful derivatives of Mahoney, P712, and Leon through neurovirulence testing in monkeys [33]. The live oral polio vaccine (OPV) was licensed in 1963 [9].

The introduction of OPVs decreased the incidence of polio by 99%, improving upon the 95% reduction achieved with inactivated polio vaccines (IPV) developed by Salk in 1955 and that were immediately used in mass vaccination campaigns [9,106,107]. The OPV has played a critical role in the Global Polio Eradication Initiative that began in 1988, decreasing the global burden of wild-type polio from more than 350,000 annual cases to just 33 cases in 2018 [108,109]. Wild-type 2 poliovirus was last seen in 1999 and declared eradicated in 2015 [110], and wild-type 3 poliovirus was declared eradicated in October of 2019 [111]. Despite these successes, any cases of polio pose a global health risk. On rare occasions, OPV can revert to a neurovirulent phenotype and induce vaccine-associated paralytic poliomyelitis, and virus excretion following vaccination can lead to circulating vaccine-derived polioviruses (cVDPVs) [9]. While cVDPVs can be controlled by consistent vaccination, long-term virus excretion by hypogammaglobulinemic vaccinees, known as immunodeficient vaccine-derived polioviruses (iVDPVs) is more difficult to manage [9]. The WHO and partners have introduced the Polio Endgame Strategy of 2019 to 2023, with the aim of wild-type and vaccine-derived polio eradication with the use of bivalent OPVs and IPVs [109].

##### Influenza

Influenza (or, more commonly, flu) is a highly contagious acute respiratory infection caused by single-stranded RNA segmented influenza viruses of the Orthomyxovirus family, which are classified into four genera known as influenza A, B, C, and D viruses [76], as well as into subtypes based on hemagglutinin (HA) and neuraminidase (NA) surface proteins [112]. Influenza affects 5–10% of adults and 20–30% of children annually across the globe [76], and predisposes individuals to secondary infections such as pneumonia [113]. Vaccination has been shown to reduce influenza mortality and morbidity, but is complicated by antigenic drift (the introduction of mutations in gene segments) and antigenic shift (the emergence of a new influenza virus subtype by the reassortment of current subtypes) [112,114]. 

Both inactivated vaccines and LAVs are currently used against the influenza virus. Trivalent inactivated vaccines (TIVs) are either whole-virus, split virus (where the virus is disrupted by a detergent), or subunit vaccines (which contain strain-specific HA and NA antigens). In contrast, live-attenuated influenza vaccines (LAIVs) are cold-adapted reassortant vaccines made with genes encoding for HA and NA proteins of the most recent seasonal or wild-type strain, and six additional gene segments from attenuated donor strains [76,112].

The influenza virus was isolated from the nasal discharge of infected patients by Wilson Smith, Sir Christopher Andrews, and Sir Patrick Laidlaw in 1933 [115]. This isolation procedure was used to develop inactivated influenza vaccines, which are grown on the chorio-allantoid membrane of embryonated eggs [116] or in cell culture [76], purified through centrifugation, and chemically inactivated [117]. Split virus and subunit inactivated vaccines followed the development of whole-virus inactivated influenza vaccines in the 1960s and 1970s [118]. These vaccines are used more than whole-virus influenza vaccines due to their reduced reactogenicity [76].

LAIVs have progressed from monovalent to trivalent and quadrivalent as new genera of influenza virus have been discovered [76,118]. The most successful and stable LAIVs are reassortant cold-adapted vaccines [119] that are passaged at progressively lower temperatures until 25 °C has been reached on primary chicken kidney cells or embryonated eggs [120,121]. The reduced growth of LAIV at higher temperatures restricts virus replication primarily to the upper respiratory tract, which mimics natural infection and prevents viral spread in the lower respiratory tract and throughout the body [76,122]. LAIVs have been used in Russia for decades with very few adverse effects other than transient febrile reactions [123], and the first LAIV to be used in the United States was a trivalent cold-adapted vaccine, which was licensed in 2003 [76]. 

Although TIVs are most frequently used for seasonal influenza vaccination [76], immunity from subunit TIVs relies on HA and NA proteins of a specific influenza strain. In contrast, the inclusion of six viral proteins of cold-adapted donor influenza strains enables LAIV to exhibit heterosubtypic protection, or to improve the viral clearance of different sub-strains of influenza than the one vaccinated for [112]. However, contraindications due to age, asthma, immunosuppression, and pregnancy often preclude the use of LAIV in comparison to TIV [76]. An improved understanding of LAIV attenuation would assist with the development of new influenza vaccines that provide longer-lasting immunity and protect against multiple strains of influenza.

### 2.3. Investigating the Molecular Mechanisms Governing LAV Attenuation

The high potency of LAVs is well established. However, their attenuation and immunogenicity originated through a purely empiric process rather than from rational engineering and development. Whether a LAV was isolated from a related pathogen and propagated in various ways, or attenuated through serial passage, the environment in which a pathogen is grown influences its evolutionary path and therefore alters aspects of its infectious cycle. For this reason, the origin of vaccinia, the basis of the smallpox LAV, is lost to history. Prior to the eradication of smallpox, attempts to recreate a vaccinia-like LAV through the passage of the variola virus major or minor in embryonated eggs, tissue culture, and mice, were unsuccessful [124]. Additionally, even when methods of LAV passage are described in detail, as in the case of YFV-17D [32], recreation of the vaccine is not always possible. The future of LAV generation, therefore, cannot be empiric; it must stem from a well-developed understanding of the differential host–pathogen interactions LAVs and virulent pathogens’ display. 

Direct genetic comparisons between LAV strains with their virulent counterparts have been used to hypothesize correlates of attenuation and immunogenicity. For example, cases of vaccine-derived paralytic poliomyelitis (VAPP) and increased neurovirulence are associated with mutations in the 5’ noncoding region of all three OPV strains after passage in the human gastrointestinal tract [125,126]. Interestingly, besides vaccinia virus-derived smallpox LAV and deliberately reassortant LAIV, LAVs tend to show high genetic similarity to the pathogen they were derived from. YFV-17D and YFV-Asibi only diverge by 68 nucleotides (about 0.6% of the genome) and 32 amino acid changes that are scattered throughout the genome [127]. Additionally, Edmonston-derived MV-LAV have been shown to differ from the Edmonston strain at most by 0.3% [128], and the RD1, a 9.5 kb deletion seen in all BCG LAVs [129], accounts for only very little of the 4,345,492 bp genome of *M. bovis* [130]. Genetic variation between LAV strains can, however, complicate extrapolation from these data. In addition to the 9.5 kb RD1 deletion, further attenuated BCG LAV strains can have additional deletions, namely RD2 (10.7 kb) and/or RD3 (9.3 kb) [129]. 

Description of genetic differences alone can hardly explain the effects they have on host–pathogen interaction and immune regulations. Therefore, a large body of research on molecular mechanisms governing LAV attenuation has been conducted in vitro. LAVs have, for instance, been found to differ in cell entry mechanisms in comparison to their virulent counterpart. Enhanced CD46 receptor utilization by MV-LAV has been described and is influenced by mutations in the hemagglutinin (H) protein [131,132]. YFV-17D and YFV-Asibi also exhibit distinctive usages of cell entry pathways and cell surface molecules, which were associated with differential viral spread and cell–intrinsic-immune-response induction [133,134]. Differences in viral replication and cytokine induction have also been observed in cell culture. YFV-17D replicates more extensively than YFV-Asibi in hepatoma cell lines [135,136,137]. However, YFV-Asibi causes a stronger and more sustained pro-inflammatory response than YFV-17D [138,139,140] in primary human vascular endothelial cells, Kupffer cells, MDMs and monocyte-derived dendritic cells (MoDCs). The Sabin type 3 OPV displays genetic differences from poliovirus type 3 that decrease the binding of polypyrimidine tract-binding protein (PTB) at its viral internal ribosome entry site (IRES). Lower PTB binding to Sabin type 3 OPV IRES impaired protein translation in neurons, and was suggested to mediate viral attenuation in the central nervous system [141]. Finally, reduced YFV-17D replication in dendritic cells (DCs) in vitro has been suggested to promote antigen presentation in the lymph nodes (LN) by preventing DC apoptosis [142,143]. Unlike YFV-Asibi, the replication of YFV-17D in MoDCs also stimulates IFNγ production in CD4+T cells [140]. Another interesting concept for explaining LAV attenuation relies upon the genetic stability of these pathogens. For instance, YFV-17D is poorly prone to diversify into multiple quasi-species in comparison to YFV-Asibi, and YFV-Asibi diversification is not associated with the emergence of attenuation mutations [144]. A more recent study reported that YFV-17D has a stronger resistance to mutations, and suggested that YFV attenuation could be related to a higher fidelity of the YFV-17D replication complex [145]. 

Although in vitro models have been useful in our understanding of LAVs, modelling host–LAV interactions in a spatiotemporal manner and in a relevant tissue context remains fundamental to accurately investigating the molecular mechanisms governing LAV attenuation and immunogenicity. To this end, animal models have been used to conduct research in vivo. For instance, RD1 deletion of BCG LAVs was reported to prevent the cytolysis produced by early secretory antigenic target (ESAT-6) and decreases tissue invasiveness in mouse models [146]. Mice defective for type I interferon signaling have been employed to explore the mechanisms of YFV-17D attenuation and immunogenicity. Humoral and CD4+ T cell responses, but not the CD8+ T cell responses, were found to be critical for YFV-induced protection [147]. IFN-γ responses were also reported to restrict the dissemination of YFV-17D, but not YFV-Asibi, in mice [148,149]. Beyond mice, NHPs have also served to investigate the impact of cell-culture adapted mutations on viral virulence and inflammatory responses. The passage of a pathogenic wild-type MV (Davis87-wt) in Vero cells and chicken embryo fibroblasts led to the emergence of several adaptive mutations that resulted in attenuated clinical symptoms in rhesus macaques [150]. Interestingly, a back-to-back comparison of early transcriptomic responses during YFV-17D and -Asibi infection was conducted in rhesus macaques. The number of differentially expressed genes in peripheral mononuclear cells was higher upon YFV-Asibi than YFV-17D infection, and expression changes were mostly connected with the dysregulation of immune responses and apoptosis. In contrast, gene expression changes during YFV-17D infection were mainly linked to innate immunity [151]. 

Although agreement has been found between in vivo and in vitro studies, discrepancies have also arisen. Sabin type 1 OPV is attenuated in vitro upon exposure to high temperatures (>37 °C), unlike its parental strain, Mahoney. However, genetic determinants of temperature sensitivity do not contribute to viral attenuation in transgenic mouse models [152,153]. Additionally, while most NYCBH-derived LAV strains examined by Lee et al. replicated to wild-type levels in vitro, their replication in mouse epithelium differed greatly from wild-type vaccinia virus, and their immunogenic effect was dose-dependent [154]. Other categories of discrepancies have also been reported. For instance, the route of administration seems to affect virus protein roles during vaccinia virus infection [155], or BCG-induced immune responses in NHPs [156]. Moreover, multiple studies have found greater IFN production in MV-LAV strains [157,158,159,160,161], but the potential presence of 5’ copy-back defective interfering (DI) RNAs in laboratory virus stocks, which increase interferon (IFN) and interferon-stimulated gene (ISG) levels (among multiple other effects), may have confounded previous results [162]. Disparities between in vivo models have also been observed. Mice defective for type I interferon signaling have been suggested to be a relevant system to investigate YFV-17D-induced protective immunity [147]. However, an important number of genes upstream and downstream of the type I interferon signaling pathways are upregulated upon YFV-17D vaccination in humans [163]. Furthermore, although the authors reported that mouse CD8+ T cells do not contribute to protection in mice defective for type I interferon signaling, multiple studies have described the critical mobilization of human CD8+ T cells toward the robust effector and memory phenotypes during YFV-17D vaccination in human vaccinees [164,165,166,167]. Finally, discrepancies have also been observed in the cytokine response of human and rhesus macaques MoDCs upon YFV-17D infection [140]. 

LAV attenuation is a multi-faceted and complex topic. The history of LAV passage is varied and mosaic, and the efficacious products we are left with are near impossible to trace to their roots. Our understanding of LAV attenuation appears to be mainly restricted to either genetic changes upstream of the vaccination process, or to immune correlates downstream of vaccination (such as antibody production). However, the immunological mechanisms and host–pathogen interactions that govern attenuation during vaccination remain uncharted. To explore these mechanisms, the choice of the right experimental system is of critical importance, as is exemplified by the instances of disagreement described above. An accurate understanding of LAV-induced immunity is therefore fully reliant on a suitable in vivo model, able to recapitulate human immunity to LAVs. A more comprehensive (although not exhaustive) summary of LAV attenuation mechanisms is provided in Table 2, and is associated with the following additional references: [168,169,170,171,172,173,174,175,176,177,178,179,180,181,182,183,184,185,186,187,188,189,190,191,192,193,194,195,196,197].

## 3. The Quest for Suitable and Cost-Effective in Vivo Systems to Investigate LAVs

### 3.1. Of Mice, Men, and Non-Human Primates

Our limited understanding of the mechanisms of LAV attenuation and immunogenicity mostly originates from the lack of in vivo systems suitable for investigating the anti-LAV immunity. Although in vitro studies have shed light on the characteristics of some LAVs (see Section 2.3), LAV attenuation and immunogenicity is defined by specific interactions with different tissues and complex cell populations over time and space, and these interplays remain impossible to model in cell culture dishes. 

Mouse models have been truly transformative for our understanding of human immunology and will very likely continue to be [198]. Although they have served as a model organism to investigate anti-LAV immunity and evaluate LAV candidates in vivo [134,146,147,148,149,153,154,155,183,184,193,194,199,200,201], important limitations and concerns have stalled their use for this purpose. Despite sharing many physiologic and metabolic processes, mouse and man diverged evolutionarily more than 96 million years ago [202]. This evolutionary divergence has differentially shaped the architecture of the immune system of both species, therefore leading to important differences in how each organism mobilizes its immune system to fight a given disease [203]. There are numerous immunological differences between mice and humans, such as differences in immune cell composition in the blood, differences in the spatiotemporal expression of many immune proteins and receptors (pattern recognition receptors, cluster of differentiation, immunoglobulin, Fc receptors, Major Histocompatibility Complex (MHC) etc.), differences in hematopoiesis, and differences in cytokine function [203]. As we aim to investigate anti-LAV immunity at the highest possible resolution, translating immunological mechanisms from mice to humans becomes perilous. Moreover, many viral and bacterial pathogens display a very narrow host tropism, often being restricted to humans and specific primate species. For example, the natural resistance of mice to viral pathogens such as MV, YFV, dengue virus, Ebola virus or Zika virus adds an additional layer of limitations to conventional mouse models. 

NHPs have been useful resources for studying anti-LAV immunity as well as evaluating novel LAV candidates. Especially, macaques have been intensively used to evaluate the immunogenicity of LAV strategies targeting human immunodeficiency virus 1 (HIV-1). However, HIV-1—like several other human-tropic viruses—is a virus that replicates poorly in NHP species. Therefore, many LAV strategies have been evaluated in NHPs using live-attenuated simian immunodeficiency virus (SIV) [204,205,206,207,208,209], the simian version of HIV-1 that only replicates in NHP species. Additional strategies employing HIV-1 related viruses [210,211] or engineered viruses harboring HIV-1 or SIV antigens [212,213] have also been tested in NHPs. NHPs have also been employed to investigate and/or evaluate the immunogenicity of many other LAVs, such as viral vectors harboring Ebola virus glycoproteins [214,215,216,217,218], LAIV [195], OPV [189,190], MV-LAV [150,185,197,219,220,221], BCG [156], live-attenuated Venezuelan equine encephalitis virus [222], YFV-17D [151] or live-attenuated Zika virus [43]. NHP models for LAV research, however, present significant limitations. First, a pathogen with a strictly restricted human tropism imposes the use of surrogate pathogens and/or a host environment that is not naturally permissive to the human pathogen that is targeted. Second, NHP studies are costly, require complicated logistics, and adequate reagents that are of limited availabilities. Finally, whether NHP cell-intrinsic innate immune responses differ from those of humans during antigen stimulation is unclear and may depend on the experimental context [140,223,224]. Therefore, although it is certain that NHPs represent an invaluable system for LAV investigation and development, they need to be complemented with more cost-effective and accessible models that allow for the monitoring of human-specific immune responses and human–pathogen-specific interactions. 

Major insights into anti-LAV immunity have been obtained from cohorts of human vaccinees. For instance, studies have identified specific cellular, transcriptomic and epigenetic signatures defining LAV immunogenicity in the blood of human vaccinees [164,166,225,226,227,228], providing unique insights into how the immune response is mobilized upon LAV vaccination in humans. However, these studies fall short of probing fundamental mechanisms that govern LAV attenuation and immunogenicity for several reasons. First, human cohort studies are often restricted to the analysis of the peripheral immune response. The upstream immunological events that occur at the site of vaccination and in draining lymph nodes, and with them the critical pathogens–host interactions that prime the immune system toward a specific direction, remain inaccessible in human vaccinees. Second, capturing key immunological mechanisms and regulations that define immunogenicity require the performance of a back-to-back comparison of the immune responses induced by a given LAV and the virulent strain it is derived from or related to. However, for obvious ethical reasons, this is not possible in a cohort of human patients. Finally, more and more studies have provided evidence that age, immunological history and microbiota status can influence immune responses to pathogens and vaccines. Therefore, identifying the immunological regulations that define a specific immune response to an LAV, independently of inter-individual variations that may impact such responses, remains a very complex task.

Altogether, the limitations of the mouse, NHP and human models highlight the need for alternative in vivo systems that would successfully combine the advantages of each models while lessening the limitations. Mice engrafted with human tissues, and especially with components of the human immune system, nicely fit such a definition. 

### 3.2. Human Immune System Mouse Models 

Per definition, humanized mice can be referred to as mice engrafted with human tissues and/or expressing human genes. Here, we will focus on mice engrafted with components of a human immune system, or HIS mice, and describe the past and current models that are of relevance to studying LAV-induced immunity.

#### 3.2.1. Development of Mouse Strains for Human Hematopoietic Stem Cell Engraftment

The story of HIS mice began with the development of immunodeficient mouse strains. In 1983, a mouse harboring the severe combined immunodeficiency (SCID) mutation, a mutation in the *Prkdc* gene, was reported to lack functional B and T cells through defective V(D)J recombination [229]. In 1992, mice defective for expression of the two recombination activating genes, *RAG1* and *RAG2,* were also reported to display no functional T and B cells through a similar mechanism [230,231]. In the late 1980s, the transplantation of adult SCID mice with fetal human hematopoietic stem cells (HSC) and tissues [232] or peripheral blood leukocytes, led to the differentiation of human T and B cells in vivo [233]. Human HSC engraftment of bg/mu/xid mice was also reported at the same period. These animals were athymic and had lower levels of natural killer (NK) cells and lymphokine-activated killer cells (LAK). Intravenous inoculation of human bone-marrow-derived cells into adult irradiated bg/mu/xid mice led to the proliferation and differentiation of HSCs into macrophage progenitors in vivo [234]. Engraftment in these animals led to a higher number of progenitors than in SCID mice, likely due to the absence of NK and LAK activity in bg/mu/xid mice. 

A few years later, adult irradiated SCID mice (CB17-SCID) transplanted with human bone marrow cells and treated with multiple human cytokines (namely, erythropoietin, human mast cell growth factor, or with a fusion protein of human interleukin-3 and human granulocyte-macrophage colony stimulating factor) could be repopulated with differentiated human cells deriving from several myeloid and lymphoid lineages in the bone marrow [235]. However, engraftment levels still remained low. In 1995–1996, SCID mice built within the non-obese diabetic (NOD)-background were identified to support higher level of HSC engraftment in comparison to previously tested genetic backgrounds [236,237,238]. Around ten years later, the underlying mechanism of this superior engraftment was found to be related to a polymorphism in the *Sirpa* allele [239]. NOD SIRPα, a cell surface receptor expressed at the surface of macrophages and encoded by the *Sirpa* allele, displays significant similarities with the human one in comparison to those expressed by mice of other genetic backgrounds. This similarity allows NOD mouse macrophages to bind to human CD47 and, therefore, to recognize human hematopoietic cells as ‘self’. 

Another important step in the development of the permissive mouse genetic background for human HSC engraftment went through the development of mouse defective or carrying a mutation into the common gamma chain of the Interleukin (IL)-2 receptor (IL2Rγ) [240]. IL2Rγ is a receptor for multiple cytokines such as IL-2, 4, 7, 9, 15 and 21. Disruption of IL-15-mediated signaling induces severe deficiencies in NK cell development and maturation, leading to a more severe immunodeficiency and more permissive environment for human HSC engraftment in adult *RAG2^-/-^* supplemented or unsupplemented by the exogenous administration of human cytokines [241,242], and in adult *NOD-SCID* mice [243]. Another important step that followed was the demonstration that the intrahepatic injection of human CD34+ cells into newborn *Rag2^−/−^ IL2Rγ^null^* and *NOD-SCID IL2Rγ^null^* mice led to the induction of low, albeit significant, functional adaptive responses in these mice [244,245], which opened the door to novel investigations of human immunity in vivo. An alternative model to the *NOD-SCID IL2Rγ^null^* mice, namely *NOD-SCID JAK_3_^null^,* has also been reported. In this model, *JAK_3_*, a gene encoding for a tyrosine kinase critical for IL2Rγ-mediated signaling is knocked-out instead of *IL2Rγ*, resulting in a complete lack of B, T and NK cells [246].

**Figure 1:**
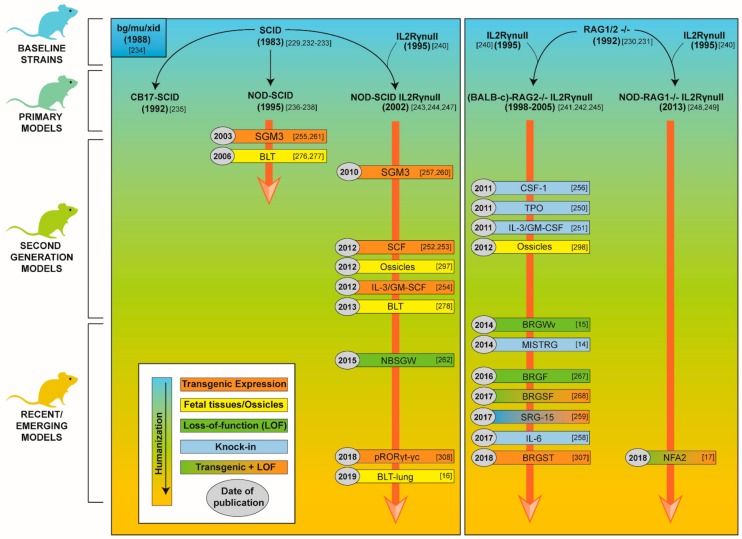
Development of human immune system mice. Schematic illustration displaying the different human immune system mice (HIS mice) developed since 1988. Models are classified into four categories that are color-coded: baseline strains (blue), primary models (blue-green), second generation models (green), and recent/emerging models (green-orange). Models are organized from top to bottom according to their level of humanization and the time when they were first developed. The year where each model was first developed (gray area), as well as the reference numbers (# under bracket) connecting to the literature that described such model, are indicated. Each second generation and emerging model is displayed in a colored box that indicates the nature of the enhancement (transgenic expression, tissue engraftment, loss-of-function (LOF), knock-in or transgenic expression+LOF). BLT, Bone-marrow Liver Thymus mice; BRGF, BRG*-Flk2^−/−^*; BRGSF, BRG *-Sirpa^tg^ Flk2^−/;^* BRGST, BRG *- Sirpa^tg^ TSLP^tg^*; BRGWv, BRG-Kit*^Wv/Wv^*; CSF-1, Colony stimulating factor-1; GM-SCF, Granulocyte-macrophage colony-stimulating factor; IL2Rγ, interleukin-2 receptor gamma chain; IL3, interleukin-3; IL-6, interleukin-6; MISTRG, BRG-*CSF1^h/h^ IL-3/CSF-2^h/h^Sirpa^tg^ TPO^h/h^ RAG2^−/−^ IL2Rγ ^−/−^*; NBSGW, NSG*-Kit^w41^*; NFA2, NRG- Flk2^−/−^HLA-A2*0201^tg^; NOD, non-obese diabetic; pRORγt-γc, NOG-pRORγt-γc; RAG, recombination activating genes; SCF, stem cell factor; SCID, severe combined immunodeficiency; SGM3, stem cell factor, granulocyte-macrophage colony-stimulating factor, and interleukin-3; SRG-15, BRG *-Sirpa^tg^ RAG2^−/−^ IL2Rγ ^−/−^ IL-15^tg^*; TPO, thrombopoietin.

As of now, the vast majority of mouse strains used for human HSC engraftment still very much rely upon the mutations and polymorphism described above. The most commonly used strains are the *NOD-SCID* [236,237,238], the *NOD-SCID-IL2Rγ^null^* (NSG or NOG, harboring a with *NOD/LtSz* or *NOD/Shi* background respectively) [243,244,247], the *BALB-c-RAG2^−/−^IL2Rγ^null^* (BRG) [241,242,245] or the *NOD- RAG1^−/−^IL2Rγ^null^* (NRG) [248,249]. Figure 1 summarizes the development timeline of these models.

#### 3.2.2. Second Generation Humanized Mouse Models and Emerging Models

Despite significant improvements in HSC engraftment, developing HIS mice on the NSG, NOG, BRG or NRG background able to mount potent innate and antigen-specific human immune responses remained a considerable challenge by the mid-2000s. Indeed, these strains bear important limitations that restrict the development and functionality of a human immune system such as (i) lack of proper human cytokine environment; (ii) absence of large niche space; (iii) limited myelopoiesis; (iv) lack of specialized human microenvironments and (v) lack of human MHC. Therefore, considerable efforts have been undertaken in the last decade to generate novel xenorecipient strains overcoming these multiple limitations. The development timeline of the models described below is shown in Figure 1.

Cytokine Environment and Niche Space

One approach to enhance HIS mice has notably consisted of generating xenorecipient strains expressing human cytokines. A large numbers of mouse cytokines do not cross-react with human receptors, and the absence of cytokine-mediated signaling has been hypothesized to affect many arms of the immune system in HIS mice, such as myeloid and NK cell development, T cell education and B cell maturation. Consistently, many groups have reported the development of BRG (with transgenic expression of human Sirpa or not), NSG and NOG mice expressing one or several human cytokines through transgenic or knock-in approaches, such as human thrombopoietin (TPO), IL-3, IL-15, granulocyte-macrophage colony-stimulating factor (GM-CSF), stem cell factor (SCF) and/or macrophage colony stimulating factor (M-CSF) [14,250,251,252,253,254,255,256,257,258,259,260,261]. These models showed enhanced human hematopoiesis as well as the superior development and differentiation of multiple myeloid cell subsets. One of these models, named MISTRG (BRG*-CSF1^h/h^IL-3/CSF-2^h/h^Sirpa^tg^ TPO^h/h^ RAG2^−/−^ IL2Rγ ^−/^^−^*), combines the knock-in of human thrombopoietin, IL-3, GM-CSF and M-CSF, and displays enhanced innate immunity against viral and bacterial infection in comparison to NSG mice [14]. 

An alternative approach to promote enhanced human hematopoiesis and myeloid cell development has involved increasing the stem cell niche space for human hematopoietic lineages to develop. In NSG, NOG, NRG and BRG mice, murine myelopoiesis is not restricted, therefore closing-up the niche space required for proper human myelopoiesis. Additionally, the presence of a (semi)-functional mouse myeloid compartment can also interfere with the initiation of human-like innate responses in HIS mice hence, introducing bias into the human immune responses that are under analysis. An initial strategy to enhance niche space in humanized mice has consisted of irradiating newborn or adult immunodeficient mice prior to engraftment. However, this approach can have negative long-term effects on animal health. More recently, NSG and BRG mice harboring a mutation in the gene coding for c-KIT/CD117 (respectively, NSG-*Kit^w41^*/NBSGW and BRG-*Kit*^Wv/Wv^/BRGWv) were generated [15,262]. c-KIT is a receptor expressed at a high level at the cell surface of HSC and progenitor cells and c-KIT-mediated signaling has been shown to regulate hematopoiesis [263]. Interestingly, adult non-irradiated NSBGW and BRGWv mice demonstrated robust and sustained engraftment of human HSC at a similar level to irradiated NSG mice [15,262] following the injection of cord blood-derived human CD34+ cells, underscoring that human the stem cell niche space can be significantly opened without the need for irradiation. 

Fms like tyrosine kinase 3 (FLT3), or fetal liver kinase-2 (Flk2), are cell surface receptors broadly expressed on early hematopoietic precursors in the bone marrow. Myelopoiesis is impaired in Flk2-deficient mice (*Flk2^−/−^*) [264,265] and injection of the human version of the ligand of FLT3 (hFLT3LG) has been shown to promote dendritic cell development in NOD/SCID mice [266]. However, hFLT3LG cross-react to mouse FLT3, allowing for dual expansion of the mouse and human myeloid compartment in conventional immunodeficient strains. Recently, Flk2-deficient NRG, BRG and BRG-*hSirpa* mice (NRGF, BRGF and BRGSF, respectively) expressing—or injected with—hFLT3LG were reported to promote the selective expansion of human dendritic cells and natural killer cells following human HSC engraftment [17,267,268]. The strong myeloid development in these HIS mice was likely the result of an effective synergy between an increased myeloid niche space caused by the Flk2 deficiency and a robust hFLT3LG-mediated signaling in human cells. 

Finally, it is worth noting that expressing human cytokines through a knock-in approach into HIS mice can also represent a great strategy to opening niche spaces. Indeed, such an approach can concurrently promote the development and maturation of human hematopoietic lineages and deplete the mouse hematopoietic compartment of the essential cytokines required for its proper development and function. 

##### Human Microenvironment and Lymphocyte Education

B- and T-cells are primarily educated in the bone marrow and thymus, respectively (the primary lymphoid tissues), where they undergo several rounds of positive and/or negative selection [269,270]. This process, which is fundamental for proper lymphocyte maturation, antigen reactivity and specificity, is regulated by extensive cross-talk between immature lymphocytes and a large variety of cell types, molecules and members of the MHC residing in primary lymphoid tissues. 

A initial approach has been to generate HIS mice expressing transgenic MHC molecules to educate human T cells in the mouse thymus and to track human antigen-specific T cells upon microbial challenge [271,272,273,274]. However, a more elaborate strategy has involved the construction of HIS mice engrafted with human primary lymphoid tissue in order to more physiologically model human hematopoiesis and lymphocyte education. In 1990, an introductory study reported that the co-engraftment of small fragments of the fetal liver and thymus into adult SCID mice (also referred as the SCID-hu model) led to the long-term maintenance of several human hematopoietic progenitors and to sustained human T lymphopoiesis [275]. About 16 years later, HIS mice co-engrafted with human HSCs and small pieces of fetal liver and thymus (namely bone marrow-liver-thymus mice or BLT mice) were generated using the NOD/SCID background [276,277], and later in the NSG background [278]. In these studies, BLT-HIS mice were shown to display enhanced lymphopoiesis and T-cell selection on human MHC molecules. BLT-HIS mice have notably constituted a model of choice to investigate the infectious cycle of HIV-1 in vivo, as mucosal infection, persistent viremia and cellular responses—albeit limited—could be recapitulated in this model [279,280,281,282,283,284,285,286,287]. BLT-HIS mice have also been a useful system to investigate the replication dynamics of the Dengue virus (DENV) in vivo, as well as anti-DENV adaptive immunity [288,289,290,291,292]. However, a major limitation of the BLT model is its absolute reliance on fetal tissues. Therefore, alternative approaches involving the engraftment of immunodeficient mouse strains with embryonic stem cell-derived, thymic epithelial progenitors [293], or with neonatal thymus [294], have been recently proposed. 

The bone marrow is critical for the survival and maintenance of HSC, as well as B-cell education and maturation. Several groups have reported the development of HIS mouse models (using mainly NSG and NSG-derived strains) engrafted with bone marrow-derived mesenchymal stromal cells (predifferentiated or not) leading to the formation of humanized ossicles in vivo [295,296,297,298,299,300,301,302,303,304]. In these studies, the subsequent injection of HSCs into the ossicles or intravenously resulted into the development of a human microenvironment able to recapitulate human bone marrow morphology and function in vivo, as well as a more physiologically relevant human hematopoiesis. 

Another major caveat in immunodeficient mouse strains is their lack of functional draining lymph nodes (LN). *IL2Rγ^null^* immunodeficient mice are defective for IL-7-mediated signaling, which results into the absence of lymphoid tissue inducer cells (LTi) during development [305,306]. Interestingly, the thymic stromal lymphopoietin (TSLP) shares a structural and functional homology with IL-7 but signals through a receptor that is IL2Rγ independent. It has been recently shown that TSLP overexpression compensates for the lack of IL-7 signaling and promotes robust LN development in BRG-*hSirpa* mice (BRGST) [307]. Even though such secondary lymphoid structures cannot be completely considered a human microenvironment per se, mouse LNs were effectively repopulated with human hematopoietic cells in BRG mice [307]. They provided a suitable niche for human T- and B- cell priming after immunization, leading to enhanced human antigen-specific responses. An alternative approach for restoring mouse LN has also been reported [308]. NOG mice harboring a transgene coding for murine IL2Rγ under the control of the endogenous promoter of RORγt were generated (NOG-pRORγt-γc), allowing the selective re-expression of IL2Rγ in a specific LTi lineage [308]. As with BRGST mice, this model was able to mount enhanced antigen-specific IgG responses. 

#### 3.2.3. Engraftment Protocols and Variables

Beyond the genetic background and modifications of a given HIS mouse, multiple protocols of engraftment have been described and can significantly impact human immune reconstitution. Engraftment protocols are composed of several variables which mainly include: the source of HSC, engraftment of fetal tissues (or not), mouse age, transplantation route, sex, and conditioning [11]. Although some engraftment protocols are widely used across several strains of HIS mice and can interrogate many biological processes, other protocols (which, for instance, include the engraftment of additional human tissues or matrices) can be more specific to certain strains and biological questions. Therefore, in addition to the HIS mouse strain that is used, it is important to understand the characteristics of the engraftment protocol that is employed to make sure that it will be appropriate for a given investigation. 

Human HSCs can be isolated from the fetal liver or from an adult donor, the latter including various sources such as cord blood, adult-mobilized peripheral blood or adult bone marrow [11]. HSCs can be either injected into newborn mice (1–4 days old, through intravenous, intrafemoral, intrahepatic, or intracardiac routes) [14,17,245,307] or into adult mice (4–12 weeks of age, through intravenous, intrafemoral, intrasplenic, or intraperitoneal routes) [15,276,277,299]. Human peripheral blood lymphocytes can also be directly injected into adult HIS mice through the intrasplenic, intravenous or intraperitoneal route [11,309,310,311]. Interestingly, sex has also been reported as an engraftment variable in some specific experimental settings [312]. 

The number of injected cells can vary from 10^3^ to 10^6^ cells per mice, depending on the model that is used [14,15,16,17,232,233,234,235,236,237,238,241,242,243,244,245,246,247,248,249,250,251,252,253,254,255,256,257,258,259,260,261,262,267,268,271,272,273,274,275,276,277,278,279,280,281,282,283,284,285,286,287,288,289,290,291,292,293,294,295,296,297,298,299,300,301,302,303,304,307,308,309,310,311,312]. Given the limited availability of HSCs, it is very common that a single (or very few) donors are used to generate several cohorts of HIS mice. Although this likely allows experiments to overcome interindividual variation and generate more robust datasets, the lack of donor diversity can also represent a limitation by overrating the significance of some of the biological aspects investigated in these models. 

To open-up niche space and decrease graft rejection, newborn or adult mice can be preconditioned prior engraftment through sublethal irradiation [14,17,245,307] or antibody-pretreatment [313,314,315]. However, HIS mouse models, capable of reaching high levels of human immune reconstitution without the need of preconditioning, have been recently described [15,262]. 

Human immune reconstitution takes, on average, between 8 and 12 weeks following engraftment, and the lifespan of HIS mice can range widely, from six months to up to 20 months [316], depending on the protocol and genetic background of the model that is employed. Indeed, several HIS mouse models can be prone to graft-versus-host disease (due to the priming of allogeneic T cell responses by murine myeloid cells) or anemia (due, for instance, to human macrophages’ activity against murine red blood cells).

As mentioned in Section ‘Human Microenvironment and Lymphocyte Education’, engraftment protocols can also include the engraftment of additional human tissues or matrices, which includes: fetal or neonatal thymus, fetal liver or bone-marrow-derived mesenchymal stromal cells (among other cells/tissues). Thymus or liver tissues are commonly engrafted under the kidney capsule, prior to the injection of autologous HSC (through the intravenous route) [279,280,281,282,283,284,285,286,287] or not [275]. For the development of humanized ossicles, bone-marrow-derived mesenchymal stromal cells can be engrafted subcutaneously by either direct injection [299] or surgical implantation [298]. Autologous HSC can be then subsequently injected through an intravenous [296] or intraossicle route [299].

## 4. Contributions of HIS Mice to LAV Research

**Figure 2:**
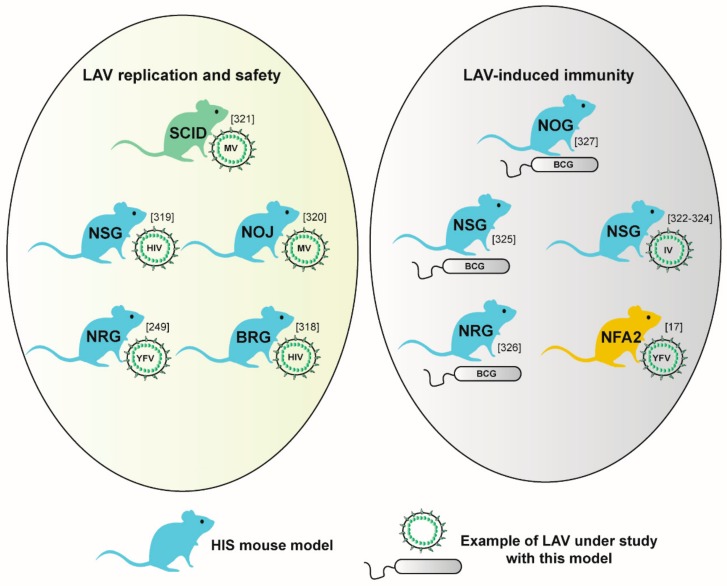
Use of human immune system mice for live-attenuated virus research. Schematic representation of the past contributions of human immune system mice (HIS mice) to investigate live-attenuated vaccines (LAV) replication and safety (left) and LAV-induced immunity (right). Each model is defined by its acronym and is color-coded similarly to Figure 1. LAVs are associated with the model they have been investigated with, and are codenamed as follows: MV, live-attenuated measles virus; HIV, live-attenuated human immunodeficiency virus; YFV, live-attenuated yellow fever virus; IV, live-attenuated influenza virus; BCG, Tuberculosis Bacille Calmette-Guérin vaccine. For each model and LAV, a reference number (# under bracket) connecting with the associated literature is indicated. *BRG*, *BALB-c-RAG2^−/−^IL2Rγ^null^;* NFA2, NRG- Flk2^−/−^HLA-A2*0201^tg^; NOG*, NOD/shi-SCID-IL2Rγ^null^;* NOJ, *NOD-SCID JAK_3_^null^;* NRG*, NOD- RAG1^−/−^IL2Rγ^null^;* NSG, *NOD/LtSz-SCID-IL2Rγ^null^;* SCID, severe combined immunodeficiency.

HIS mice have the potential to truly transform our understanding of LAVs. They can provide unique opportunities to investigate the molecular basis governing the high immunogenicity of currently established LAVs, and therefore shed new light onto our understanding of human immunity and vaccine design. Additionally, they can also present a cost-effective way to evaluate the replication fitness, immunogenicity, attenuation and safety of LAV candidates in a human immunological context. Here, we will review how conventional and emerging models of HIS mice have been harnessed to investigate LAV replication fitness, safety and immunogenicity. A summary of the contributions of HIS mice to LAV research can also be found in Figure 2. 

### 4.1. Assessing LAV Replication Fitness and Safety in HIS Mice

HIS mice have been intensively used to investigate the HIV-1 infectious cycle in vivo and immune responses [317]. However, very limited studies have employed these systems to evaluate the efficacy and safety of attenuation strategies for HIV-1. One study reported a HIV-1 strain (HIV-rTA) for which the TAT-Tar mechanism required for virus replication and transcription was inactivated and the viral protein Nef was replaced by a doxycycline-dependent transcription system [318]. Authors showed that HIV-rTA could lead to production infection in doxycycline-fed BRG-HIS mice without CD4+ T cell depletion. Importantly, the virus was not found to escape doxycycline control over time and revert toward a wild type TAT-tar mechanism [318]. A few years later, a second version of the same virus expressing a ubiquitin-processed rTA-Ub-Nef cassette (HIV-rTA-Ub-Nef) was found to replicate more effectively in NSG-HIS mice than HIV-rTA without impacting the CD4+ T cell count [319]. Altogether, these studies demonstrate that HIS mice represent a useful system to evaluate the replication kinetics and safety of live-attenuated lymphotropic viruses that have a very narrow tropism. 

Humanized *NOD-SCID JAK_3_^null^* (NOJ) mice have been shown to be susceptible to MV-LAV infection [320]. This is especially evident in studies where MV-LAV could be significantly detected in the blood, spleen and bone marrow of the humanized NOJ, but not in non-humanized NOJ [320]. HIS mice’s susceptibility to MV-LAV has been used to evaluate vaccine reversion and stability over time. Specifically, CB17-SCID-hu mice were employed to investigate whether long-term passages of an avirulent measles vaccine strain (Moraten strain) could cause enhanced virulence and thymic tissue adaptation over time [321]. Interestingly, the Moraten strain, collected 90 days post vaccination from the thymus of CB17-SCID-hu mice, showed high virulence in thymus implants in comparison to the original Moraten strain. These works demonstrate that HIS mice can represent a valuable resource to evaluate vaccine safety. 

We recently reported that NRG mice engrafted with human HSC (NRG-HIS) are susceptible to YFV-17D [249]. Indeed, although NRG mice engrafted with murine HSCs (NRG-MIS) were not permissive to infection, the reconstitution of NRG mice with human hematopoietic cells led to persistent infection in the peripheral blood and in the spleen, demonstrating a preferential tropism of YFV-17D for human cells. By tracking viral RNA into multiple subsets of the human immune system, we were able to probe the cellular tropism of YFV-17D over time and space in multiple human hematopoietic lineages. Using NRG-HIS mice and mice depleted for Stat1 signaling in the hematopoietic compartment, we also identified species-specific interactions between YFV-17D and this compartment that potentially regulate the outcome of infection. Altogether, this work provided important evidence that HIS mice can serve as a unique system to define the dynamics of interactions between LAVs and components of the human immune system over time and space. 

### 4.2. Investigating LAV-Induced Immunity in HIS Mice

Live-attenuated influenza vaccines (LAIV) likely represent the LAVs that have been the most investigated using HIS mice. In 2008, Yu et al. reported the use of NSG-HIS mouse models engrafted with HLA-A*0201 HSC, then treated with hFlt3L and reinjected with autologous T cells prior to vaccination with a trivalent influenza LAV. Following vaccination, LAIV-CD8+ T cells targeting an HLA-A*0201-restricted epitope from the influenza matrix protein (FluM1) and nonstructural protein 1 were found to expand in the blood, spleen and lungs, underscoring the successful adaptive immune priming in this model [322]. Importantly, priming was reported to be myeloid-dependent, which highlights the critical need of a myeloid compartment in HIS mice for modeling anti-LAV immunity. In a follow-up study, the same authors identified that CD1c+ dendritic cells induce the differentiation and accumulation of mucosal effector CD103+ CD8+ T cells in a TGFβ-dependent manner in the lung following LAIV vaccination [323]. Finally, in a third publication, they identified CD141+ dendritic cells as the major driver of the establishment of the Th2 response in the lung upon LAIV infection, by promoting the differentiation of IL-3- and IL-4-producing CD4+ T cells through the OX40 ligand [324]. Overall, these studies demonstrate that HIS mice can be successfully employed to uncover immunological mechanisms regulating T cell priming and differentiation in relevant tissues during LAV infection.

NSG-HIS mice have been reported to mimic clinical features of human tuberculosis infection [325]. Following aerosol infection, pulmonary lesions and necrotic granulomas displaying similarities with those observed in patients could be observed. Authors also demonstrated the relevance of such an infection model to evaluate drug regimens against M. *tuberculosis* in vivo. In another study, NRG-HIS mice were immunized subcutaneously with the BCG vaccine and then challenged four weeks later with M. *tuberculosis* via the respiratory route. BCG was able to partially protect NRG-HIS mice against M. *tuberculosis infection*, as demonstrated by a reduced bacterial load and granulomatous lesions in the lungs. The authors therefore concluded that HIS mice can represent a relevant platform to evaluate novel vaccinal strategies against M. *tuberculosis,* a method that they further implemented by testing a novel adenoviral-vectored (VV) vaccine expressing an immunodominant M. *tuberculosis* antigen [326]. The same year, an additional study from a different group reported the analysis of CD4+ and CD8+ T cell phenotypes (through their expression of IFNγ, TNFα and IL-2) in NOG-HIS mice upon *M. tuberculosis* infection, as well as BCG vaccination prior to M. *tuberculosis* infection [327]. These authors found evidence of potential T-cell activation upon M. *tuberculosis* challenge in the lungs, and further suggested that BCG vaccination affects the distribution of T-cell activation phenotypes in the same tissue upon subsequent M. *tuberculosis* infection. However, unlike the previous study, authors did not find that BCG could confer any level of protection against M. *tuberculosis* in NOG-HIS mice, in contrast to C57BL/6 mice and Hartley guinea pigs, where partial protection was observed [327]. Altogether, these studies highlight that HIS mice have potential in uncovering the molecular basis of BCG-induced immunity. However, they also underscore the need for more advanced HIS mouse models able to more accurately and reproducibly recapitulate human BCG-induced immunity. 

### 4.3. Emerging Models for LAV Research

Previous studies using conventional HIS mice have demonstrated the importance of the myeloid compartment in HIS mice for accurately investigating anti-LAV immunity. More recently, we reported NRGF-HIS mice (see Section ‘Cytokine Environment and Niche Space’ for model description) and NRGF-HIS mice expressing transgenic HLA-A*0201 (NFA2-HIS) as a new HIS mouse model for investigating YFV-17D-induced immunity [17]. In this model, the adenovirus-mediated expressing of hFLT3L promotes the selective expansion of human dendritic cells, myeloid cells and granulocytes, unlike in conventional NRG, NSG or BRG mice, where hFLT3L expression/injection leads to the expansion of both the human and murine myeloid compartment. Although this might not be an issue for all LAVs, the mouse interferon response has been shown to strongly restrict YFV-17D infection in mouse models of infection [148,328,329], in contrast to humans, where YFV-17D can overcome such a response. Therefore, hFLT3L-mediated expansion of the myeloid compartment in conventional HIS mouse strains can lead to important immune interference between the mouse and human hematopoietic compartment. Unlike conventional NRG-HIS mice (in which the human myeloid compartment is not expanded), we found that NFA2-HIS mice can mount a peripheral transcriptomic response upon YFV-17D that shared key similarities with the response observed in human vaccinees [17]. NFA2-HIS mice were consistently able to mount a YFV-17D-specific CD8+ T cell response as well as an YFV-17D-IgG response, which led to the clearance of viral RNA in the peripheral blood, unlike in conventional NRG-HIS mice where infection persist over time in periphery. This work demonstrates the strong potential of second-generation HIS mice and emerging models to uncover fundamental immunological processes regulating adaptive immune priming in vivo and in a human context, and therefore sheds light on the elusive mechanisms governing LAV-induced protective immunity.

## 5. HIS Mice and LAV: Limitations and Future Opportunities 

Although HIS mice have been instrumental to investigating the infectious cycles of important viral and bacterial pathogens [12,13,330,331], the use of these models to study established and candidate LAVs has remained scarce. 

One potential reason explaining this scarcity of this research is the current limitations of these models. Unlike virulent pathogens that often display low immunogenicity features, evade or impede the immune responses and lead to clinical symptoms, LAVs are strongly immunogenic pathogens that requires an in vivo system powerful enough to effectively ‘read’ them and harness them in order to develop a strong and long-lasting response. Therefore, although conventional HIS mice have been considered satisfying enough to study specific immunological processes during virulent pathogen infection, investigating LAV-induced immunity requires models able to mount polyfunctional, potent and long-lasting responses that involve multiple arms of the innate and adaptive immune system. However, conventional HIS mice, like NOD-SCID, NRG, NSG/NOG or BRG, display major caveats such as low myeloid engraftment, a lack of important lymphoid tissues and the absence of a proper lymphocyte education, therefore making these models poorly suitable to investigate LAV-induced immunity.

While HIS mice are still a work in progress and will likely always be, models that have been emerging over the past five years represent a promising step forward in the development of better HIS mice for LAV research. For instance, models with enhanced myeloid and NK engraftment, such as the MISTRG [14], NSG-SGM3-HIS [257,332] or NFA2-HIS [17], have been reported to display superior T and/or B cell activity upon microbial challenges than conventional models. Specifically, the NFA2-HIS mice provided a demonstration that enhancement of the human myeloid and NK compartment could promote a superior innate and adaptive immune response to an LAV in HIS mice [17]. 

Nevertheless, major challenges need to be overcome to establish models able to mount strong and sustained T and B cell responses that would resemble those observed in humans. Recently, we reported a new methodology to quantitatively evaluate the quality of the human immune response in HIS mice by defining a correlation index between the peripheral transcriptomic responses measured in a given HIS mouse and in humans following YFV-17D vaccination [17]. Although we were able to show that NRG-HIS had a significantly lower correlation index than NFA2-HIS mice, the correlation index of this latter model was found to reach no more than 0.18. This result highlights that major refinements are still urgently needed in second generation HIS mice to allow for an accurate modeling of the human immune responses to LAV. Back-to-back comparative studies of these responses in humans and HIS mice will be of critical help to rationally guide the design of novel models and evaluate the benefits of specific requirements.

**Figure 3:**
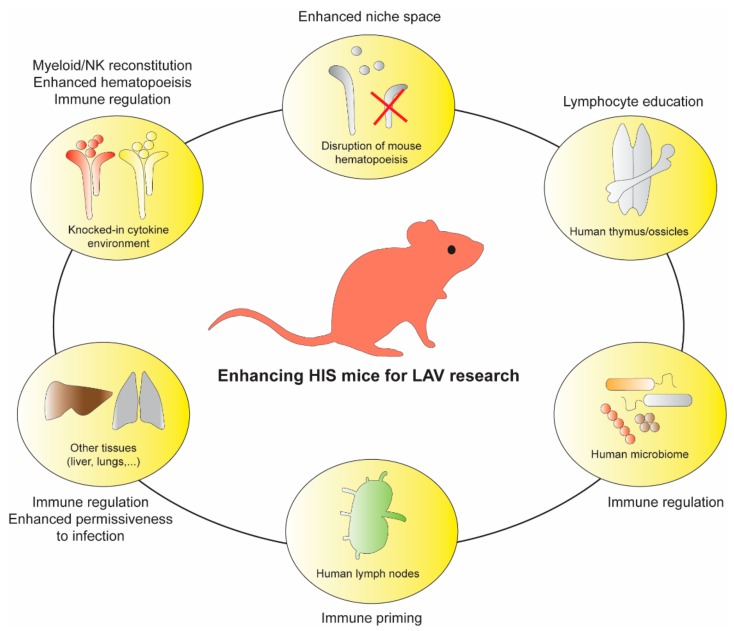
Enhancing human immune system mice for LAV research. Schematic representation of key improvements and immunological synergies required for building the next generation of human immune system mice (HIS mice), and foster live-attenuated vaccine (LAV) research and development.

Given the importance of the myeloid and NK compartment in priming adaptive immune responses, one area of improvement relies upon enhancing the human cytokine environment of the most recent models through the combined knock-in of multiple cytokines such as IL-6, IL-15, Flt3LG, TPO, IL-3, GM-CSF and M-CSF [14,250,251,252,253,254,255,256,257,258,259,260,261]. The co-engraftment of novel cytokine-enhanced HIS mice with human HSC, human thymus and/or ossicles would provide an optimal environment for lymphocyte education, maturation and priming and would be highly relevant for LAV research. Models combining superior myeloid engraftment (via human cytokine knock-in), mouse MHC knock-out and the transgenic expression of a large panel of human MHC class I and II could also represent a suitable alternative (Figure 3). 

A large number of LAVs (as well as other vaccines) are injected intra-muscularly and therefore prime the immune response in draining LN. However, current HIS mice used for LAV research lack functional secondary lymphoid structures because of defective IL7-mediated signaling (See section ‘Human Microenvironment and Lymphocyte Education’). This suggests that immune priming in HIS mice upon LAV infection is either extremely limited when using an intra-muscular route, or occurs in irrelevant tissue context when using other routes of infection (priming will mostly occur in the spleen if using an intravenous route of infection). Several strategies have been suggested and/or reported to generate HIS mice with LN which include, among other cytokine-mediated restorations of mouse LN [307,308], engraftment of ex-vivo generated human artificial LN [333], or direct engraftment of human LN [334]. HIS mice models combining functional LN structures, primary lymphoid human tissues or human MHC class I and II expression, as well as an enhanced myeloid-NK compartment could represent a fantastic resource for LAV research (Figure 3). Such models would open major avenues to a new ranges of studies where human immunological mechanisms governing LAV immunogenicity and attenuation could be decrypted in a relevant tissue and cellular context, and in a way that is not possible in human vaccinees. 

Other human tissues could enhance LAV-induced immunity in HIS mice. Conventional HIS mouse strains co-engrafted with human liver and human HSC have been previously reported and were shown to be a relevant system to study, for instance, hepatitis B virus infection replication and induced-immune responses [335,336]. The liver is known to host several immune functions such as IL-6 production, secretion of acute phase proteins, and the production of complement or Kupffer-cell mediated immune regulation [337]. Therefore, it is likely that the immune function of the liver could synergize with those of the immune system when co-engrafted in refined HIS mouse models such as the one described above (Figure 3). 

More recently, BLT-HIS mice engrafted with human lungs have been reported [16]. Authors showed that human lung tissues could be repopulated with autologous human hematopoietic cells, and that mice could mount humoral and T-cell responses following microbial challenge. Human lung tissues could be of relevance when investigating immune responses to LAVs administrated through the respiratory route, such as LAIV, for instance (Figure 3).

Finally, more and more evidence points to the role of the microbiota in regulating immune responses to vaccines [338]. A recent study conducted in human vaccinees notably found that antibiotic treatment impairs IgA and IgG responses to H1N1 following influenza vaccination [339], suggesting that particular bacterial species can modulate adaptive responses to vaccines. HIS mice present a significant advantage over humans for the study of the microbiota as their microbiome can be easily tracked and manipulated throughout the animal’s life. Protocols have been recently developed to generate HIS mice engrafted with human microbiota [340], and few preliminary studies have been conducted in conventional HIS mouse models [341]. As we approach the role of the microbiota in regulating human health, the development of innovative HIS mouse models able to mount superior adaptive responses and co-engrafted with human microbiota would open notable opportunities to investigate LAV-induced immunity in a more relevant physiological context and to better understand microbiota-mediated regulation of immune priming (Figure 3). 

## 6. Concluding Remarks

LAVs undeniably represent one of the most important biomedical innovations in human history. Yet, much remain to be learned from these vaccines. The molecular basis of their attenuation and potent immunogenicity are still poorly understood, which consequently restricts our fundamental understanding of human immunity. LAVs represent formidable tools to better understand human immune responses and how they are regulated, and could not only provide us with effective new ways to rationally design innovative vaccines and immunotherapies against challenging infectious diseases, but also to treat other human pathologies, such as cancer.

HIS mice have been instrumental in investigating the infectious cycle of many human pathogens. However, despite their strong potential, their applicability to LAV research has remained limited, at both the fundamental and pre-clinical (e.g., the testing of LAV candidates) level. This is mostly due to the limited ability of past and current models to mount a sufficiently strong and accurate immune response to these highly immunogenic pathogens, therefore pushing investigations toward less relevant (mouse) or more costly and logistically challenging (NHP) animal models.

Although HIS mice will never be able to perfectly mimic human immune responses, recent model improvements have demonstrated that creating HIS mice able to mount more potent responses to LAV is possible. In the future, efforts will have to be directed toward the generation of HIS mice that can mount potent polyfunctional immune responses tightly regulated by different arms of the innate and adaptive immune system. The design and objective evaluation of models combining functional lymphoid structures with enhanced human cytokine environments, erythro-myeloid physiological engraftment and additional human-specific immunoregulators (such as the liver or microbiota) could be central to this endeavor.

It is worth noting that recapitulating the establishment and persistence of memory B and T cell responses in HIS mice during vaccination will remain a considerable challenge. Nevertheless, the accurate modeling of the immunological events occurring from the time of vaccination to the activation of the adaptive response in secondary lymphoid tissues should be seen as a reachable objective. These set of events are likely critical in defining the potent immunogenicity of successful LAVs, and capturing them could definitively impact our understanding of LAV-induced immunity, and human immunity overall. 

## Figures and Tables

**Table 1:** Comparison of commonly used vaccine types.

Vaccine Type	Immunogenicity	Reactogenicity	Safety and Stability
LAV	+++	+++	+
Inactivated	++	++	++
Subunit	+	+	++
Toxoid	+	+	++

Qualities including the immunogenicity, reactogenicity, and safety and stability of vaccine types are compared. The immunogenicity of live-attenuated vaccines (LAVs) is superior to that of other vaccine types, although they are more reactogenic. Although the risk is extremely low, LAVs have the potential to revert to a virulent phenotype, therefore their stability is lower than inactivated, subunit, and toxoid vaccines. Reactions to other vaccine types may also occur, but the risk is lower than for LAVs. +++: high; ++: moderate; +: low.

**Table 2:** LAV history and non-exhaustive list of attenuation mechanisms.

LAV	History		Attenuation Mechanisms		
LAV Type	Method of Generation	Date of Isolation/ Attenuation	Genetic Similarity to Parental/Virulent Strain	In Vitro Findings	Animal Models
Smallpox	Isolation of related pathogen	1798 [25]	n/a (vaccinia vs variola virus)	[154,171,172,173,174]	Mouse [154,155]
Bacille–Calmette Guerin (BCG)	Attenuation of related pathogen	1921 [29]	>99.7% (RD1 deletion from *M. bovis*) [129,130]	[175,176,177]	Mouse [146,193]
Yellow fever virus (YFV)	Attenuation of causative pathogen	1937 [32]	99.37% [127]	[133,134,135,136,137,138,139,140,142,143,144,145,178]	Mouse [134,147,148,149]; NHP [151]
Measles	Attenuation of causative pathogen	1954 [34]	≥99.7% [128]	[131,132,157,162,179,180,181,182,183,184,185]	Mouse [183,184]; NHP [185,197]
Poliovirus	Attenuation of causative pathogen	1954 [33]	≥99.9% [168,169,170]	[141,152,153,186,187,188,189,190]	Mouse [153]; NHP [189,190]
Influenza	Attenuation of causative pathogen	1965, 1967 [120,121]	n/a (reassortant vaccine)	[191,192]	Mouse [194]; NHP [195]; Pig [196]

Historical aspects of live-attenuated vaccine (LAV) creation, such as method of generation, as well as the date of the pathogen’s isolation or attenuation are listed. Mechanisms of LAV attenuation are categorized into (i) genetic similarity to their parental strain or a virulent strain (with the exception of smallpox and influenza LAVs), (ii) studies on mechanisms governing LAV attenuation performed in vitro, and (iii) animal models used for in vivo investigation. NHP, non-human primates; n/a, non-applicable. References linking to the associated literature are indicated in brackets.
